# The Promise and Challenges of Bioprinting in Tissue Engineering

**DOI:** 10.3390/mi15121529

**Published:** 2024-12-23

**Authors:** Ryan Martin, Daeha Joung

**Affiliations:** 1 Department of Physics, Virginia Commonwealth University, Richmond, VA 23284, USA; martinr12@vcu.edu; 2 Massey Cancer Center, Virginia Commonwealth University, Richmond, VA 23298, USA

Organ transplantation, biomimetic organ models, and the restoration of damaged or eviscerated tissues have been key goals in surgical and medical research since their inception [1,2,3,4]. However, the shortage of suitable donors and the challenges of achieving sufficient biocompatibility with current treatment options often result in patients succumbing to diseases and injuries [5,6]. Advances aimed at creating fully functional biomimetic synthetic organs and tissues hold the potential to address these pressing needs, paving the way for rapid, personalized surgical solutions. Bioprinting has emerged as a leading field in these efforts, enabling groundbreaking progress to be made in treating tissue- and organ-related conditions [5,6,7,8,9,10,11]. Given the diverse structural and functional complexities of tissues and organs within living systems, bioprinting employs various strategies to replicate different areas of the human body [7,8,9,10]. As highlighted in the journal *Micromachines*, researchers have successfully modeled tissues and organoids, including those of the skin, liver, urinary tract, brain, kidneys, heart, and lungs. These achievements utilize techniques such as extrusion-based, laser-guided, coaxial, and multi-material bioprinting [12,13,14,15]. Many of these advancements result from interdisciplinary collaboration across fields such as systems, chemical, and biological engineering, diagnostic radiology, pathology, biophysics, and developmental biology. These collective efforts have contributed to the development of numerous biotechnologies and materials currently the subject of clinical trials and further research [16,17,18]. Despite these strides, bioprinting in tissue engineering remains constrained by challenges such as the limited functionality of biomaterial composites, insufficient print resolution, and the inability to precisely regulate the biological cascades governing tissue development and organ maturation [19,20]. Additionally, financial and regulatory barriers continue to hinder progress, delaying transformative discoveries in developmental biology and medical applications [21].

As a general overview, bioprinting involves designing scaffold-based or scaffold-free architectures that serve as substrates to support and guide cellular proliferation [22,23,24,25]. In scaffold-based approaches, natural and synthetic biomaterials such as hydrogels and decellularized extracellular matrix (ECM) composites are combined with scaffolds to encapsulate living cells, providing essential nutrients and nanoscopic environments for cell attachment during deposition [26]. The mechanical properties of cell-laden hydrogels and synthetic biomaterials are critical for maintaining print fidelity, which depends on the materials’ fluidic and native environmental characteristics during deposition [20,27,28,29]. Following deposition, these materials typically require a curing process activated by ionic gradients, photopolymerization, temperature changes, or enzymatic reactions [20,30]. With the utilization of these manufacturing processes, comprehensive studies on the history, methodologies, and physical dynamics of bioprinting, in addition to the biomaterials and crosslinking agents frequently employed, address many key chemical and biological considerations in these platforms [20,26,27,28,30,31,32,33,34,35,36,37,38,39,40].

Extrusion-based bioprinting is currently the most widely adopted technique in tissue engineering due to its ability to deposit fluid materials layer by layer, effectively mimicking the micro-scale structures of native tissues and organs. To date, the only clinically approved application of bioprinting is the implantation of a bioprinted ear for reconstructive surgery, though its long-term efficacy remains under investigation [41,42]. Moreover, ongoing research has advanced the development of tissue and organ models for the skin, heart, bone, cartilage, liver, lungs, pancreas, nervous system, and vascular system. These models have improved scientific understanding of targeted drug efficacy, facilitated reconstructive treatments for chronic diseases, and identified challenges in achieving complete system fabrication [15,16,43,44,45,46,47]. The broad range of potential applications for 3D bioprinting is well documented in various studies, particularly those focused on tissue and organ research [41,43,44]. Among them, 4D bioprinting has emerged as a promising innovation, offering temporal control over cellular interactions and the ability to change shapes into intricate native structures dynamically [19,47,48,49,50,51,52]. This approach overcomes current limitations in print fidelity, enabling detailed analyses of multi-cellular, layered, and time-sensitive interactions observed during organismal development and maturation.

Challenges in replicating biomimetic constructs, ranging from proteins to organs, are primarily driven by limitations in biomaterial compatibility and functionality [25,43,53,54]. These challenges are frequently the result of the multi-functional characteristics of native ECM and cell components that govern the processing and/or production of specific signaling molecules, in addition to cell-to-cell and cell-to-environment communication. Key mechanical properties of cells, such as surface topology, porosity, hydrophilicity, and the sourcing of synthetic biomaterials, must also be carefully considered to ensure appropriate biocompatibility within specific biological contexts [20,55]. These multi-faceted requirements for biomaterials are what make synthesizing new composites that are non-toxic and biocompatible challenging. Even when such properties are achieved, batch-to-batch variations can significantly impact the desired genetic expression, as cells respond to physical cues in their microenvironments, activating signaling cascades to adapt to or modify the surrounding matrix composition [56]. Biomaterial limitations also include constraints in print resolution and cell viability. Current bioprinting techniques typically achieve consistent accuracy at scales of ~100 μm while maintaining low shear rates to protect cells, which remains insufficient for replicating native microenvironments [20,25]. Furthermore, the intermediary biological cascades that govern cell maturation, senescence, and tissue formation in multicellular organisms are not yet fully understood, particularly from developmental biology and biochemical perspectives [19]. These underexplored aspects are critical, as the persistent patterning observed in histological analyses is often attributed to the biophysical constants and material properties of tissues during development [57,58]. Addressing these challenges requires advancing both biomaterial science and our understanding of the intricate processes involved in tissue development and functionality.

Typically overlooked, the lack of regulatory clarity regarding the use of patient-derived cells or the procedural transplantation of lab-grown cells into a patient has also impacted advancements in bioprinting [4,21,59]. These ethical, legal, and social implications (ELSI) present challenges that must be addressed by regulatory bodies and healthcare sectors worldwide [60]. With multiple countries banning the use of animal testing for cosmetic products, demand has grown for alternatives such as bioprinting to provide biomimetic models of the skin as a replacement; however, widespread adoption of these technologies has been slow due to poor return on investment and the limited value propositions of current bioprinting capabilities [36,61]. As shown in Figure 1, a strengths, weaknesses, opportunities, and challenges (SWOC) analysis of the bioprinting field, progress by governing legislative bodies of bioprinting applications could reignite capital investment, leading to commercialization and catalyzing translational advancement in tissue engineering and broader healthcare applications [4,36,60].

Despite the aforementioned limitations, the interdisciplinary nature of bioprinting in tissue engineering presents unique opportunities for researchers to make groundbreaking discoveries, as advancements in different fields progress at varying rates. Bioprinting can be a powerful tool for investigating cellular biophysical dynamics and conducting assays such as enzyme-linked immunosorbent assay (ELISA) or real-time quantitative polymerase chain reaction (qPCR). These methods enable the identification of biochemical reactions and signaling cascades governing specific cell groups or types, thereby enhancing our understanding of intermediary and translational processes essential to organismal maturation [19,62,63]. Additionally, bioprinting facilitates the analysis of spatial, temporal, and mechanobiological factors influencing tissue integration and derivation from the perspective of group cell behavior. This process can elucidate the balance between biochemical and biophysical reactions that drive layered tissue development and regeneration [56]. Insights gained through such observations could revolutionize diagnostic radiology, illuminating cellular-level characteristics of pathological diseases and transforming medical practice through the creation of patient-derived tissue models [64,65].

There is substantial demand for the development of novel biomaterial composites. The use of artificial intelligence (AI) and machine learning (ML) to assist in the evolution of biomimetic materials that can capture all native dynamic behaviors in response to being implanted into a host and facilitate manipulation through naturally occurring inductive processes would propel global commercialization and research applications into all elements of living organisms [33,38,55,56,66]. Enhancing print resolution through robotic arm tool–path integrations and ML-assisted algorithms has already shown promise in achieving greater precision and success in constructing intricate structures [67,68]. Furthermore, a vital factor in future advancements is maintaining and ensuring high cell viability, which can be achieved through improvements in either biomaterial sourcing, which currently hinge on ethical concerns over the use and ownership of patient-derived cells, or process improvements in print head design that regulate temperature and shear stressors to which cells are introduced during printing [28,60,67,69,70].

Envisioning the next decade of bioprinting and its potential to revolutionize every aspect of interventional medicine, with collaboration between academia, industry, and government, is paramount in establishing proper regulatory frameworks that aim to move the field forward [66,69]. Over the coming decade, bioprinting holds the potential to revolutionize interventional medicine, with aspirations of implanting damaged or lost non-regenerative neural and ocular cells without relying on pluripotent or progenitor stem cells [64,71,72,73]. The future of bioprinting remains poised to transform healthcare at an unprecedented scale.

## Figures and Tables

**Figure 1:**
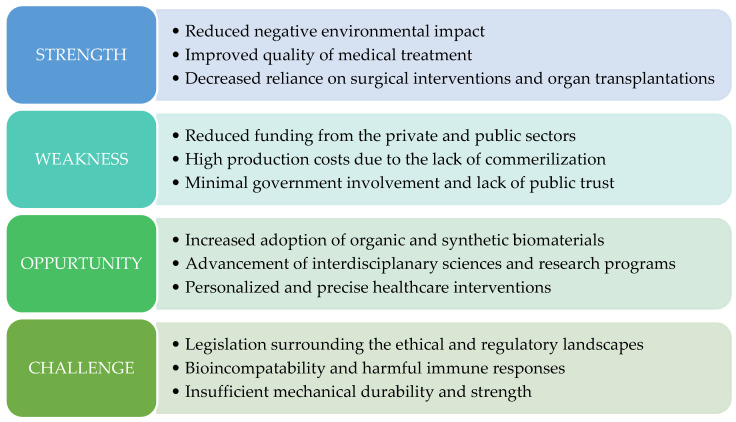
SWOC analysis of 3D bioprinting, representing the pros and cons of the field. Modified from reference [36].
